# Caliciviruses and Foodborne Gastroenteritis, Chile

**DOI:** 10.3201/eid1107.041062

**Published:** 2005-07

**Authors:** Roberto Vidal, Veronica Solari, Nora Mamani, Xi Jiang, Jimena Vollaire, Patricia Roessler, Valeria Prado, David O. Matson, Miguel L. O'Ryan

**Affiliations:** * University of Chile, Santiago, Chile;; † Health Ministry of Chile, Santiago, Chile;; ‡ University of Cincinnati College of Medicine, Cincinnati, Ohio, USA;; § Eastern Virginia Medical School and Children's Hospital of The King's Daughters, Norfolk, Virginia, USA

**Keywords:** calicivirus, diarrhea, gastroenteritis, norovirus, outbreaks, sapovirus, Norwalk, Enteric Virus

## Abstract

Human caliciviruses caused 45% of 55 gastroenteritis outbreaks occurring in Santiago, Chile, during 2000–2003. Outbreaks affected ≤99 persons, occurred most commonly in the home, and were associated with seafood consumption. Thirteen outbreak strains sequenced were noroviruses, including 8 GII, 2 GI, and 3 belonging to a novel genogroup.

Human caliciviruses (HuCVs), especially noroviruses, are a major cause of food- and waterborne outbreaks in industrialized countries. Their role as a cause of gastroenteritis outbreaks in economically developing areas is unclear because little information is available (1–3). Five norovirus genogroups have been described, with serogroup II (GII) prevailing in outbreaks worldwide since ≈1990 (3–6). Strains differing significantly from GI and GII prototypes are being increasingly reported since detection methods have improved (5,6).

Chile is a rapidly developing country. Studies have shown seroprevalence for HuCVs of >70% for children 5 years of age and incidence of 8% in acute sporadic cases of diarrhea in children (7–9). A small number of norovirus-associated outbreaks have been reported but information is scarce because no surveillance system for gastroenteritis exists (8). The capital city of Chile, Santiago, with ≈6.1 million persons, contains ≈40% of the country's population. Ninety-six public hospitals, private clinics, and emergency outpatient clinics distributed within 6 healthcare services centers are responsible for notifying the Health Ministry when infectious diseases that are on the National Mandatory Notification List are identified.

## The Study

In 1994, the Metropolitan Area Environmental Health Service (health service) began a gastroenteritis outbreak surveillance program in the centers. This program was improved in 2000 by using a standard protocol for pathogen detection. This study was to determine the role of HuCVs as a cause of gastroenteritis outbreaks from June 1, 2000, to January 30, 2003, in Santiago, Chile, by using recently improved antigen and genome detection assays, and to characterize genetically the circulating strains.

Sentinel sites were instructed to report gastroenteritis outbreaks ≤48 hours after detecting the sentinel case. A health service epidemiologist would initiate an investigation and make home visits to identify all persons possibly involved in the outbreak. Specific attack rates for implicated food products were calculated.

Stools samples for pathogen detection were collected during home visits from affected persons and were cultured for *Salmonella*, *Shigella*, *Campylobacter*, and *Vibrio* spp., according to standard techniques using selective media (10). Enteropathogenic *Escherichia coli*, enterotoxigenic *E. coli*, and enterohemorrhagic *E. coli* were studied by multiplex polymerase chain reaction (11) and enzyme-linked immunosorbent assay (ELISA). Rotavirus and enteric adenoviruses were detected by ELISA or by commercial kits (SAS Rota Test, SA Scientific Inc., San Antonio, TX, USA; Premier Adenoclone, Meridian Diagnostics Inc., Cincinnati, OH, USA; 40/41 Adeno-Strip, Coris Bioconcept, Gembloux, Belgium) and parasites were detected by Burrows technique.

All samples were tested for HuCV by a novel ELISA specific for noroviruses based on pools of sera obtained from rabbits and guinea pigs hyperimmunized with a total of 9 different norovirus capsids (12) and by reverse transcription–polymerase chain reaction (RT-PCR) targeting conserved sequences in the polymerase region of HuCVs (9). Primers used for RT-PCR were 289 (RT)/290 (PCR) or a pool of degenerate primers of last generation, 289hi for RT and 290hijk for PCR, that detect norovirus and sapovirus (13,14). RT-PCR products were cloned by using pGEM-T Easy vector system (Promega, Madison, WI, USA). The 327-base nucleotide sequences that encode for the polymerase dependent RNA were aligned by using OMIGA 2.0 (Oxford Molecular, Madison, WI, USA) software and compared with 21 prototype sequences retrieved using BLAST searches from the GenBank database. Phylogenetic distances were calculated by Kimura 2-parameter method and a phylogenetic tree was plotted by the neighbor-joining method using MEGA, version 2.1 (15). Bootstrap values were based on 1,000 generated trees.

## Conclusions

During the 30-month study, a total of 82 outbreaks affecting ≤100 persons in the Santiago metropolitan area were reported properly to the health service and investigated. In each outbreak, a rectal swab from ≥1 person was collected for microbial studies. In each of 55 outbreaks, ≥1 stool sample was collected for virus studies, and in each of 31 outbreaks, ≥1 stool sample was collected for parasite studies. Enteric microbial pathogens were isolated in samples from ≥1 person in 32% of the 82 outbreaks, and potentially pathogenic parasites were isolated in 6 (19%) of 31 outbreaks (Table 1). A total of 175 samples from 55 outbreaks were obtained for viral detection, of which 47 (27%) from 25 (45%) outbreaks were positive for HuCV by using ≥1 method. HuCV outbreaks affected ≤99 persons with a median of 5 persons (Table 1). In 16 outbreaks, ≥2 persons were positive by using ELISA or RT-PCR; in 9 outbreaks, 1 person was positive by ≥1 method. Overall, 20% of the outbreaks were detected only by ELISA, 24% only by RT-PCR, and 56% by both techniques.

**Table 1:** Proportion of acute diarrhea outbreaks associated with a bacterial enteropathogens or a human calicivirus (HuCV) and number of persons affected during the HuCV outbreaks

Year	No. outbreaks positive*/no. tested				No. affected in HuCV outbreaks
	Bacteria†	%	HuCVs	%	Range (median)
2000	8/13	61	4/12	33	3–28 (4)
2001	11/32	34	6/18	33	2–54 (5)
2002	6/34	18	14/22	64	2–99 (5)
2003‡	1/3	33	1/3	33	5
Total	26/82	32	25/55§	45	2–99 (5)

*An outbreak was associated with a given pathogen if ≥1 sample was positive. †Bacteria isolated included: enteropathogenic *Escherichia coli* (EPEC) (2), enterotoxigenic *E. coli* (ETEC) (3), enterohemorrhagic *E. coli* (3), EPEC + ETEC (1), *Salmonella sp* (12), *Shigella sp* (2), *Staphylococcus aureus* (3). ‡January 1–10, 2003. §In 1 outbreak, ETEC and EPEC and in another, Shiga toxin–producing *E. coli*, were concomitantly isolated with HuCV. In 1 additional outbreak the only pathogens simultaneously detected in 1 patient were rotavirus and adenovirus by enzyme-linked immunosorbent assay.

Most HuCV outbreaks occurred in the home, with outbreaks in childcare centers and schools occurring next most frequently; only a small fraction occurred in restaurants. The most commonly implicated food products were seafood, including raw oysters and clams (Table 2). Among a total of 1,137 persons exposed in the 25 HuCV outbreaks, 283 (25%) had typical acute gastroenteritis symptoms. Thirty-nine percent of the cases occurred in children <5 years of age, 28% occurred in children 5–14 years of age, 27% occurred in adolescents and adults 15–60 years of age, and 4% occurred in adults >60 years of age. Most commonly reported symptoms were diarrhea (86%), vomiting (36%), and fever (16%).

**Table 2:** Human calicivirus outbreak settings and implicated food products by study years

	2000–2001	2002–2003	Total (%)
No. outbreaks	10	15	25
Outbreak settings			
Home	6	11	17 (68)
Childcare center or school	2	3	5 (20)
Restaurant	1	1	2 (8)
Picnic	1	0	1 (4)
Food products implicated			
Seafood	3	11	14 (56)
Meat products	2	3	5 (20)
Prepared cooked food	2	1	3 (12)
Other	3*	0	3 (12)

*Goat cheese, mayonnaise, celery.

HuCV amplicons from 13 outbreaks evaluated belonged to the norovirus genus, including 8 GII, 2 GI, and 3 in a potentially novel genogroup. The 3 new strains differed >40% in nucleotide identity from all prototype strains compared (Figure). Bootstrap analysis based upon 1,000 generated trees yielded a node for the potentially novel genogroup in 100% of the trees. Two of the outbreaks caused by this potentially novel genogroup occurred during the same month, while the third occurred a year later. The distribution of the 8 genogroup II strains fell into 3 genetic clusters. One of the genetic clusters, represented by strain 028/10-2001, was closely related with a distance of 0.11 to Saitama virus (SaiU1, accession no. AB039775), a Japanese strain found in 1998 in a child with acute gastroenteritis. The 2 other genetic clusters are proposed as novel genetic clusters and include strains (i) O55/5-2002, O64/10-2002, O62/9-2002, O71/11-2002, O78/11-2002, and (ii) O77/11-2002, O85/1-2003 (Figure). Both clusters are also most closely related to SaiU1. The first cluster has 2 independent nodes with a distance of 0.19 to 0.28 from SaiU1, the second cluster is represented by 2 strains with a distance of 0.18 and 0.19 from SaiU1, respectively.

**Figure:**
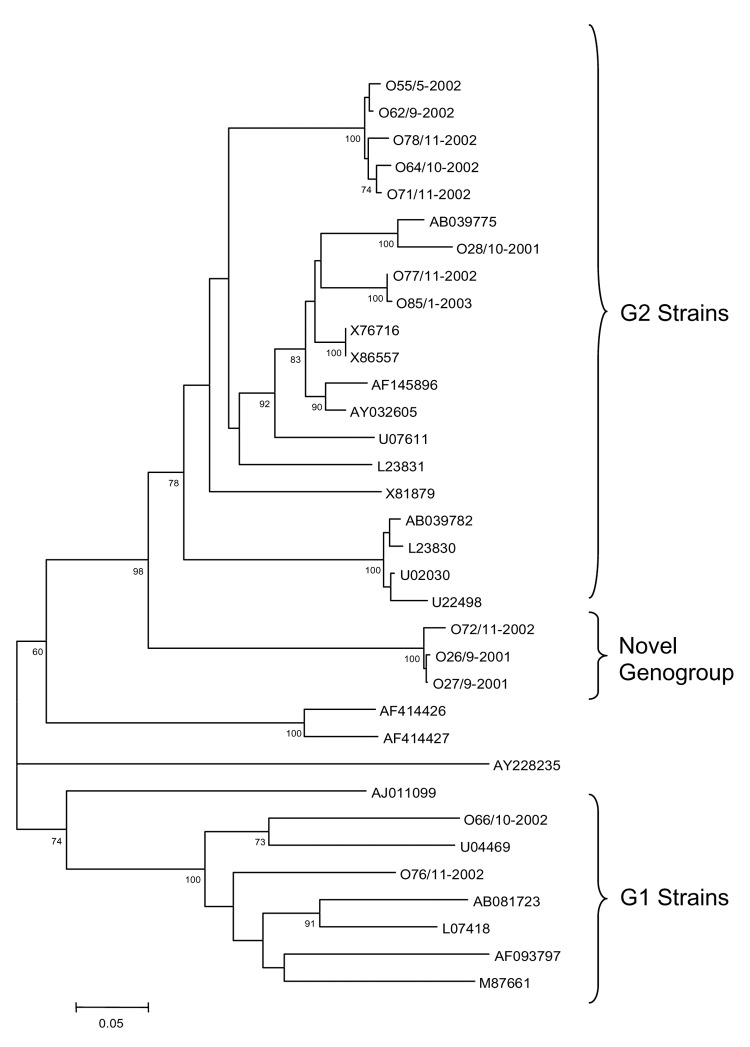
Phylogenetic tree of noroviruses based on the 327-base region of the 3´ end of the open reading frame 1 using 13 novel sequences designated according to outbreak number/month–year (example: O55/5–2002), and 21 sequences of Norwalk-like virus strains representative of the currently identified genogroups, designated according to GenBank accession number. Comparative strains include: Norwalk virus (M87661), SaitamaU1 (AB039775), Saitama U201 (AB039782), WUG1 (AB081723), Schreier (AF093797), Camberwell (AF145896), Fort Lauderdale (AF414426), Saint Cloud (AF414427), Jena (AJ011099), Maryland (AY032605), Murine NV (AY228235), Southampton (L07418), OTH25 (L23830), Snow Mountain (L23831), Toronto (U02030), Desert Shield virus (U04469), Hawaii (U07611), Mexico (U22498), Bristol (X76716), Melksham (X81879), and Lorsdale (X86557). Bootstrap values based on 1,000 generated trees are displayed at the nodes (values >60% are shown).

HuCVs were associated with almost half of 55 fully evaluated gastroenteritis outbreaks in Santiago, Chile, and were more common than outbreak-associated enteric bacterial pathogens such as *Salmonella* sp. and diarrheogenic *E. coli*. To our knowledge, this is the first prospective, active surveillance for gastroenteritis outbreaks in Latin America that included a thorough search for HuCVs. Publications from the region have described high seroprevalence for these viruses (3,16) and have reported isolated outbreaks affecting children and adults (3,8).

HuCV-associated outbreaks mostly affected children that ate seafood in homes; other implicated sources included meat products and vegetables. Estimated attack rates were ≈25%. The reported outbreaks in this study reflect the tip of the iceberg; only 10% of all reported outbreaks could be studied because of capacity and resources for prompt reporting and investigation. This study should stimulate efforts for appropriate outbreak investigation in developing regions where food products safety is important for the health of the population, tourism, and international commerce.

Genogroup II strains dominated, as in other studies (3–6), but only 1 of these strains fell into the same genetic cluster of a previously described strain, Saitama virus; in contrast, most strains grouped into 2 closely related new clusters. In addition, 3 strains, 2 temporally related, likely belong to a new genogroup. The circulation of genetically diverse strains indicates the need for further studies to understand the clinical and epidemiologic importance of such diversity.

Study funded by Chilean government grant FONDECYT 1020583-1000636.
